# Silk Fibroin as Edible Coating for Perishable Food Preservation

**DOI:** 10.1038/srep25263

**Published:** 2016-05-06

**Authors:** B. Marelli, M. A. Brenckle, D. L. Kaplan, F. G. Omenetto

**Affiliations:** 1 Department of Biomedical Engineering Tufts University 4 Colby St., Medford, MA, 02155, USA

## Abstract

The regeneration of structural biopolymers into micelles or nanoparticles suspended in water has enabled the design of new materials with unique and compelling properties that can serve at the interface between the biotic and the abiotic worlds. In this study, we leveraged silk fibroin quintessential properties (i.e. polymorphism, conformability and hydrophobicity) to design a water-based protein suspension that self-assembles on the surface of food upon dip coating. The water-based post-processing control of the protein polymorphism enables the modulation of the diffusion of gases through the silk fibroin thin membranes (e.g. O_2_ and CO_2_ diffusion, water vapour permeability), which is a key parameter to manage food freshness. In particular, an increased beta-sheet content corresponds to a reduction in oxygen diffusion through silk fibroin thin films. By using the dip coating of strawberries and bananas as proof of principle, we have shown that the formation of micrometre-thin silk fibroin membranes around the fruits helps the management of postharvest physiology of the fruits. Thus, silk fibroin coatings enhance fruits’ shelf life at room conditions by reducing cell respiration rate and water evaporation. The water-based processing and edible nature of silk fibroin makes this approach a promising alternative for food preservation with a naturally derived material.

Food waste has an impact on food quality and safety, representing a loss of economic value and resources, and, a hindrance to economic development. The Food and Agriculture Organization (FAO) of the United Nations has estimated that one-third of the food produced for human consumption worldwide is annually lost or wasted along the chain that stretches from farms to postharvest treatments, processing, distribution and end-user consumption^1^. For fruit and vegetable commodities, the FAO has estimated a 50% loss of crops throughout the food supply chain, mainly concentrated in the postharvest, distribution and end-user consumption stages and mostly due to the premature deterioration of perishable crops. Many perishable fruit and vegetables possess in fact high metabolic activity and suffer from high possibility of microbial contamination, resulting in short shelf-life, fungal decay, colour change, and off-flavour^2^. To date, several treatments have been explored to extend the postharvest life of fruit and vegetables (e.g. cryopreservation, exposure to synthetic chemical fungicides, modified atmosphere packaging, osmotic treatments, hypobaric and heat treatments)^3^. In this scenario, the formation of an edible coating represents an alternative route to extend crop freshness by combining extended storage periods with ease of handling^4^,^5^. In general, food coatings should be mechanically robust matrices with hydrophobic groups to exert low permeability to oxygen and water vapours (i.e. control over fruit respiration rate and firmness retention). Biocompatibility, biodegradability, antibacterial and antifungal activities, membrane forming capacity and safety (i.e. edible and not allergenic) should also be compelling properties for an edible coating material^6^. To date, polysaccharides, proteins, resins, lipids and their combinations are the commonly used options for coating formulations^7^,^8^,^9^,^10^,^11^,^12^,^13^. Polysaccharides and proteins are known to form films with good mechanical properties, but with poor permeability, while lipids and resins form brittle films with improved permeability. Among all the perishable food, strawberries are considered one of the most difficult to preserve fresh along the food supply chain and have therefore been used as a model to test the efficacy of food preservation strategies and, in particular, of edible coatings^7^,^14^,^15^.

Silk fibroin is an extensively investigated biomaterial for its potential in textile, biomedical, photonic, and electronic applications^16^,^17^. Silk fibroin is a structural protein, like collagen, but with a unique feature: it is produced from the extrusion of an amino-acidic suspension by a living complex organism (while collagen is produced in the extracellular space by self-assembly of cell-produced monomers). Silk fibroin properties are derived from its structure, which consists of hydrophobic blocks staggered by hydrophilic, acidic spacers^18^. In its natural state, silk fibroin is organized in beta-sheet crystals alternated by amorphous regions, which provide strength and resilience to the protein^18^. The multiplicities of forms in which regenerated silk fibroin can be processed at a high protein concentration and molecular weight make it attractive for several high-tech applications, as recently reported^17^,^19^,^20^,^21^,^22^,^23^,^24^,^25^,^26^. In addition, the amino-acidic nature of silk fibroin brings a diversity of side chain chemistries that allows for the incorporation of macromolecules useful in drug delivery applications or in providing cellular instructions^27^. The natural properties of silk fibroin have been exploited in the regenerated silk materials. The combination of beta-sheet structures with inter- and intra-molecular hydrogen bonds that provides high-flexibility in the natural fibre also guarantees extreme conformability in regenerated film format. Silk fibroin films have in fact been used as biocompatible and biodegradable substrates for implantable electrodes to be applied on the brain and as adhesive, edible sensors to monitor fruit ripening and cheese aging^21^,^22^. In addition, the gas (i.e. oxygen, carbon dioxide, water vapour) diffusion properties of silk materials present in the cocoon, which allows the *Bombyx mori* to survive through the pupal phase of its lifecycle, can be tailored in regenerated protein by regulating silk fibroin polymorphism^28^,^29^,^30^. This, for example, has ensured the correct gas exchange in silk fibroin materials engineered for corneal grafting^28^,^29^,^30^. In particular, silk fibroin with modular degrees of beta-sheet content can be obtained by regulating the time (minutes to hour range) and temperature (4–95 °C) to which the protein gets exposed to water vapour or edible polar solvents (i.e. ethanol)^26^.

Remarkably, silk fibroin has also shown to possess features that are distinct of its regenerated form. For example, regenerated silk fibroin stabilizes heat labile molecules and compounds (e.g. enzymes and antibiotics)^31^. Silk is indeed considered a platform technology in biomaterials fabrication as its robustness and qualities bring needed assets to provide a portfolio of distinct features (*e.g.* nanopatterning, biochemical functionalization) for the final construct. Processing of regenerated fibroin generally involves the partial or total dehydration of a fibroin suspension (protein content of 1–15 wt%) to form films, sponges, gels, spheres (micron- to nano-sized) and foams with numerous techniques (*e.g.* solvent casting, freeze drying, salt leaching, sonication)^32^. The rationale beyond these fabrication processes is to manufacture a robust material that combines mechanical strength with *ad-hoc* biochemical properties.

## Results and Discussion

**Figure 1:**
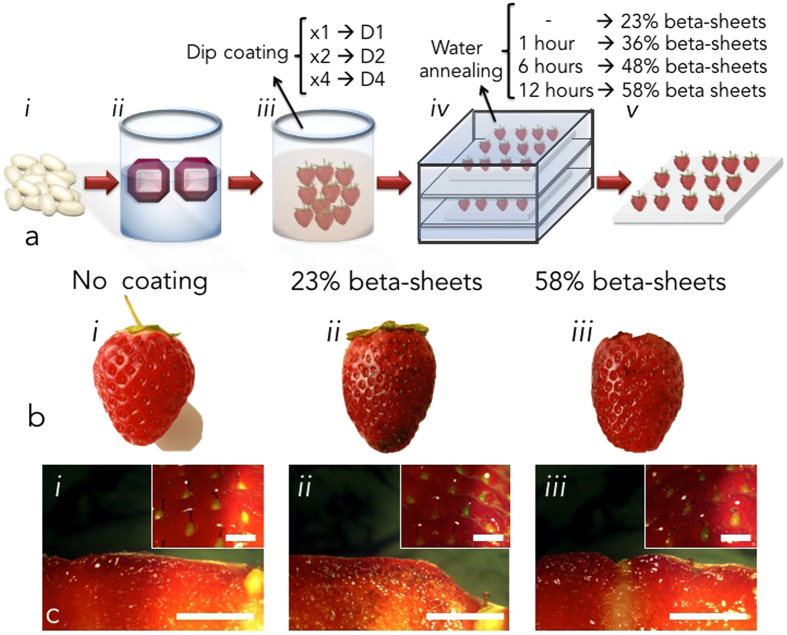
Coating of perishable fruits with edible silk fibroin. (**a**) The impact of silk fibroin coating was investigated on freshly picked strawberries. (*i*) Silk fibroin was extracted from *Bombyx Mori* cocoon fibers by dissolution in 9.3 M LiBr solution and (*ii*) dialysis in deionized water. The concentration of the protein in water was then adjusted to 1 wt%. (*iii*) Coating of strawberries was then achieved by dip coating process in silk fibroin suspension (1wt%). The dip coating process was repeated up to 4 times. *iv*) Beta-sheet content in silk fibroin edible coatings was modulated using water annealing post-processing. The longer the exposure to water vapour (up to 12 hours), the higher the beta-sheet content of the protein, as reported in Table 1. *v*) Silk fibroin-coated strawberries were then left at room conditions (T =D 22 °C, RH =D 38%) to investigate the impact of the coating on the quality of the fruit. Crystal violet dye was used to stain the silk fibroin coating. (**b**) Representative images of stained strawberries *i*) freshly picked, *ii*) coated silk fibroin edible coating (4 dip coating processes, 23% beta-sheet, *i.e.* no water annealing applied), *iii*) coated with silk fibroin (4 dip coating processes), 58% beta-sheet, *i.e.* exposed to water annealing post-processing. The crystal violet dye is barely visible on the surface of the coated strawberries (black dots) due to the few-micron thickness of the coating. (**c**) Stereoscopic images of the surface and of the cross-section (insets) of crystal violet-stained fresh strawberries (*i*) as picked, (*ii*) coated with silk fibroin with 23% beta-sheet content and (*iii*) coated with silk fibroin (4 dip coating processes) with 58% beta-sheet content. Scale bars: 2 mm.

In this study, we report the use of silk fibroin suspension as an edible coating material for perishable fruit preservation. Silk fibroin is generally considered flavorless and odorless, which are compelling properties for food coating and packaging applications^33^,^34^. A Silk fibroin suspension was used to coat freshly picked strawberries through dip coating (Fig. 1a)^32^. The molecular weight of silk fibroin was tailored in the 170–90 kDa range by regulating the boiling time (i.e. 30 minutes) during fibroin extraction and the concentration of the protein in the dipping suspension was adjusted to 1 wt% to obtain a final suspension with rheological properties (i.e. viscosity and surface tension) similar to the ones previously used for biopolymer-based coatings^7^,^35^. Inductively coupled plasma mass spectrometry (ICP-MS) was used to investigate the presence of metal elements in the silk suspension, to assess possible toxicity deriving from the silk fibroin regeneration process. For all the elements considered, the detection values were significantly below the toxicity levels in drinking water (Table S1), as per World Health Organization guidelines^36^. The coating of strawberries with silk fibroin was achieved with a two-phase process. In the first phase, a multiple-step dip coating process (number of coating steps =D 1, 2 and 4) was used to expose strawberries to silk fibroin suspension. Secondly, silk fibroin-coated fruits were treated with water vapour under vacuum (namely water annealing post-processing) to explore the effects of increasing beta-sheet content in silk fibroin materials on the management of fruits’ postharvest physiology^26^. The results suggested that silk fibroin coatings prolonged the freshness of perishable fruits by slowing fruit respiration, extending fruit firmness and preventing dehydration.

### Silk fibroin as a coating to manage strawberries’ postharvest physiology

**Figure 2:**
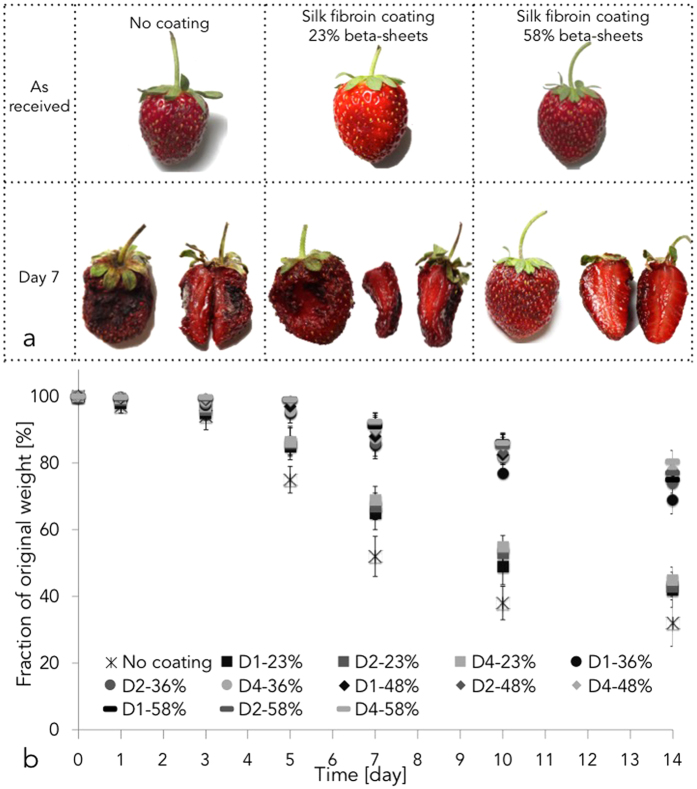
Ripening and weight loss of strawberries coated with edible silk fibroin coating. (**a**) Time-lapse of strawberries ripening. As picked strawberries were stored at 22 °C and 38% RH (no coating) or dip coated in silk fibroin suspension (23% beta-sheets coating). Water annealing was used as post-process to modulate the relative content of beta-sheets in silk fibroin. At day 7, silk fibroin coating showed to improve the quality of the stored strawberries. (**b**) Weight loss of strawberries stored for up to 14 days at 22 °C and 38% RH. Strawberries were stored as picked (i.e. *no coating*) or after coating with silk fibroin suspension (*Dx-xx%*). *Dx* stands for ‘x’ dip coating steps. *xx%* stands for relative amount of beta-sheet content. (e.g. D1-23% means that strawberries were dip coated with silk fibroin materials with 23% beta-sheets content). One-way ANOVA test with Tukey mean analysis was used to evaluate the weight loss data. Silk beta-sheet content (which is proportional to water-annealing time) but not number of dip coating steps affected the dehydration of the strawberries considered. *No coating* controls lost circa 70 wt% of their original weights in the 14 days considered (highlighted within the red rectangles). Strawberries coated silk fibroin retained more water than the *no coating* controls at day 3 (p <D 0.05). An increase in silk fibroin beta-sheets content via water annealing process further decreased fruit dehydration compared to non-water annealed counterparts (p <D 0.05) and to the *no coating* controls (p <D 0.05) but no statistically significant difference was found between samples with a beta-sheet content ≥D 36% (p >D 0.05).

The thickness of silk fibroin coatings, which was in the range of 27–35 μDm, was not statistically significantly influenced (p >D 0.05) by the number of dip coating steps (Table 1). Conversely, water annealing post-process strongly influenced the properties of the silk fibroin coatings by increasing the relative content of beta-sheet structures from 23 ±D 2% (for untreated coatings) to 58 ±D 5% (for coatings exposed to water vapour for 12 hours). The effects of water annealing time on the the relative beta-sheet content of silk fibroin coatings are reported in Table 1. No changes in the microscopic morphology of silk fibroin materials exposed to water annealing was visible with scanning electron microscopy analysis (Fig. S1). Crystal violet dye was used to stain the silk fibroin coating. Representative macroscopic images of stained strawberries uncoated and coated with silk fibroin edible materials with increasing beta-sheet content are presented in Fig. 1b *i*, *ii* and *iii*, respectively. Crystal violet staining is barely visible on the surface of the coated strawberries (black dots) due to the few-micron thickness of the coating. In addition, stereoscopic microscopy of the surface and of the cross-section of crystal violet-stained strawberries coated with silk fibroin materials showed no changes in the appearance of the fruit when compared to the uncoated control (Fig. 1c). The efficacy of silk fibroin edible coatings in managing strawberries’ postharvest physiology was evaluated as a function of number of coating steps and of silk fibroin beta-sheet content. The external and internal analyses of the aging of representative strawberries as a function of dip coating steps and of silk beta-sheet content is presented in Fig. 2a and Figure S2. A prolonged preservation of strawberries tissues corresponded to increasing coating steps and to longer water annealing process (i.e. enhanced beta-sheet content). This was visible by time dependent reduction in shading from the original red colour and deformation from the original morphology. In addition, increasing coating steps and higher beta-sheet content possibly down-regulated microbial growth, as suggested by reduced fungal- and mould-induced decay. This was also confirmed by investigation of the time dependent decay of the strawberries’ flesh. Higher beta-sheet content in silk fibroin coatings corresponded to an enhanced preservation of the internal tissue of strawberries, for the time points considered.

### Measurement of dehydration

**Figure 3:**
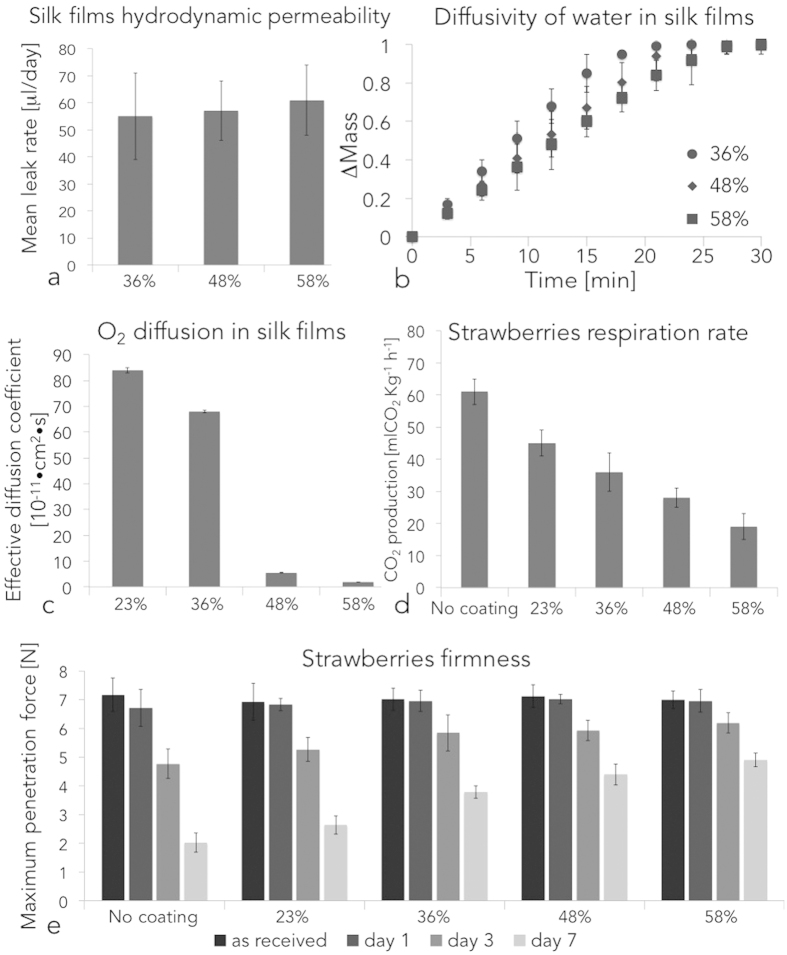
Permeability and diffusivity of water and oxygen in silk fibroin films as a function of protein crystallinity and their effects on the quality of the edible coating. (**a**) Hydrodynamic permeability of silk fibroin films. The beta-sheets content of the protein did not affect the leakage of water through the film (p <D 0.05). (**b**) Diffusivity of water in silk fibroin films. The beta-sheet content in silk fibroin materials affected the diffusivity of water in the transient state (0 <D t <D 25 minutes) but not in the steady state (t >D 25 minutes). (**c**) Oxygen permeability in silk fibroin films. Silk fibroin polymorphism strongly affected oxygen permeability as a 50 fold decreased in oxygen permeability was measured between silk fibroin with a relative beta-sheet content of 23% and 58%. (**d**) Respiration rate of silk coated strawberries was measured as a function of beta-sheet content. To higher beta-sheet contents corresponded a statistically significant decrease in the production of CO_2_ (p <D 0.05). (**e**) Effects of silk coating on the firmness of strawberries as a function of storage time and of beta-sheet content. Natural decay of the strawberries caused a decrease in the fruit firmness, as measure by a time-dependent decrease in the force required to penetrate the fruit (p <D 0.05). An increase in the coating beta-sheet content corresponded to a statistically significant delay in the decay of the fruit firmness at days 3 and 7.

Dehydration of strawberries is an indication of the breakdown of the red receptacle tissue, which results in off-flavouring, microbial decay, loss of turgor and water evaporation. Figure 2b shows the time dependent weight loss (express as fraction of original weight) of strawberries as a function of dip coating steps and of silk fibroin beta sheets content. Two-way ANOVA test with Tukey mean analysis was used to evaluate the data. Silk fibroin beta-sheet content but not number of coating steps affected the dehydration of the strawberries considered. Uncoated controls lost circa 70 wt% of their original weight in the 14 days considered. Strawberries simply coated (i.e. no water-annealing post-process) silk fibroin (Dx23%) retained more water compared to controls at day 5 (p <D 0.05). Strawberries coated with silk fibroin and further treated with water annealing process presented a decreased dehydration compared to strawberries non-treated with water-annealing (p <D 0.05) and to uncoated controls (p <D 0.05). However, water annealing time (i.e. silk fibroin beta-sheet content >36%) did not statistically significantly affect changes in the strawberries’ original weight (p >D 0.05) for all the time considered. To investigate the phenomenon of fruit dehydration through thin silk fibroin coatings, the interplay between silk fibroin thin membranes and water have been investigated (Table 2 and Table S2). In particular, hydrodynamic permeability and water diffusivity phenomena were explored (Fig. 3a,b, respectively). Investigation of hydrodynamic permeability showed that silk fibroin beta-sheet content did not statistically significantly affect (p >D 0.05) water permeation (as per one-way ANOVA test with Tukey mean analysis). The study of water diffusivity revealed that the beta-sheet content of silk fibroin membranes slightly affected mass transport of water through the protein (as per one-way ANOVA test with Tukey mean analysis). Calculated diffusion coefficients for mass transport experiments are reported in Table S2. Surprisingly, there is an apparent inconsistency between calculated diffusion coefficients for water (Table S2) and for water vapor permeability (WVP, Table 2). When silk fibroin beta-sheet content increased from 36%–58%, WVP of the film containing 58% beta-sheet content decreased to 1/100 of that with 36% beta-sheet content. However, the measured diffusion coefficient for water was only five times smaller. While further analyses are necessary to better understand these results, the data may signify that an increase in beta-sheet content induces a water vapor barrier effect without significant change of water (molecule or cluster) diffusion in the silk fibroin film. This may be due to a combination of beta-sheet domain re-arrangement during the water-annealing process (i.e. beta-sheet crystal dimension, formation of inter- and intra-molecular beta-sheet, alignment of the crystals) and by the presence of wicking phenomena.

### Gas diffusivity through silk membranes

Gas diffusivity through silk fibroin membranes plays a major role in fruit preservation as gases play a pivotal role in fruit stem cells metabolism (i.e. oxygen), are by-products of their metabolism (i.e. carbon dioxide) and may act as growth factors (i.e. ethylene in climacteric fruit). To evaluate the efficacy of silk fibroin coatings as a gas barrier, we measured oxygen diffusivity through silk fibroin membranes (t =D 70–80 μDm) with increasing beta-sheet content (Fig. 3c, Table S3). In particular, the effective oxygen diffusion coefficient was found to be modulated by protein beta-sheet content. A decrease of two orders of magnitude in the oxygen effective diffusivity coefficient was measured between silk fibroin materials with a relative beta-sheet content of 23 ±D 5% (no water annealing treatment) and 58 ±D 4% (12 h of water annealing).

Respiration rate is an important parameter to evaluate the metabolic activity of the stem cells present in the fruits. The higher the respiration rate, the higher the metabolic activity and the faster the fruit decay. In this study, the respiration rate of silk fibroin-coated strawberries was measured as a function of beta-sheet content in silk materials (Fig. 3d). To higher beta-sheet content corresponded a statistically significant decrease in the production of CO_2_ (p <D 0.05), which indicated a decrease in the respiration rate of the fruits. It was evident that the beta-sheet content of the edible silk fibroin coatings controlled the gas exchange of between the strawberries and the environment due to the strong influence that silk polymorphism has on the permeability to gases like CO_2_ and O_2_. The increase in beta-sheet content, in fact, yields a less hydrated material (decrease in free water and freezing bound water) where the molecules are more packed and organized in nanoscopic beta-sheet structures^26^,^37^. O_2_ and CO_2_ are not soluble in silk proteins, while they are soluble in water. It is then possible that the dissolution of gas decreases for silk materials that have a decreased amount of water (i.e. higher beta-sheet content) in their structure, resulting in a decreased diffusion of water-soluble gases through silk membranes.

### Firmness rate in silk fibroin-coated strawberries

A puncture (or penetration) test was also used to evaluate the effects of silk coating on the firmness of strawberries as a function of storage time and of beta-sheet content (Fig. 3e). While natural decay of strawberries caused a decrease in the fruit firmness, as measured by a time-dependent decrease in the force required to penetrate the fruit (p <D 0.05), an increase in the coating beta-sheet content corresponded to a statistically significant delay of fruit firmness decay at days 3 and 7.

### Silk fibroin as coating for climacteric fruits

**Figure 4:**
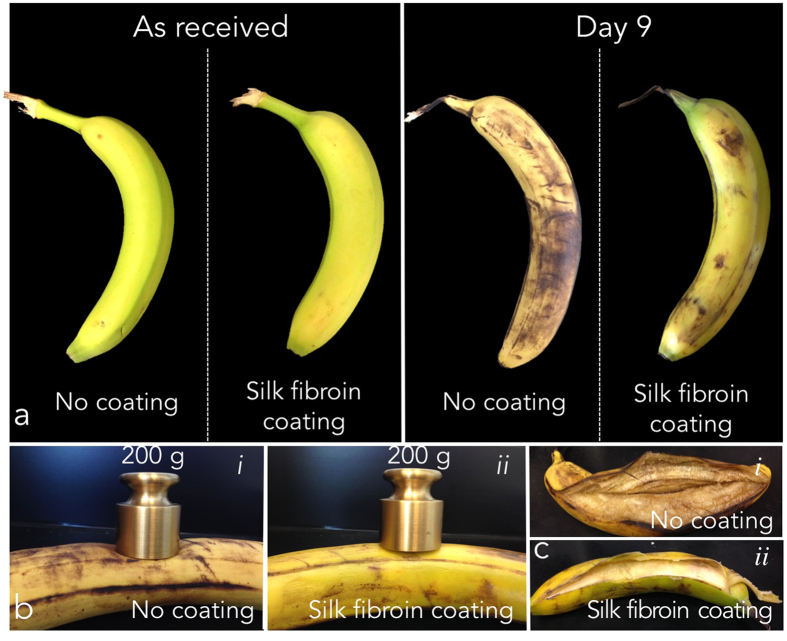
Evaluation of bananas ripening with and without crystalline silk coating. Fruits were stored at 22 °C and 38% RH as received (*no coating*) and after coating with silk fibroin materials. Bananas were hanged from their respective stem throughout the whole experiment. (**a**) Time lapse photography of banana ripening indicating that silk fibroin coating decreased the ripening rate. (**b**) Investigation of silk fibroin-coated banana turgidity (i) when compared to uncoated controls. Turgidity was studied qualitatively by applying a dead load (200 g) on the surface of the fruit. (ii). The test was accomplished ad day 9 after coating. Silk fibroin-coated banana showed an increased firmness, when compared to uncoated control. (**c**) Images of the internal flesh of non-coated (i) and silk fibroin-coated (ii) bananas at day 9 post coating-treatment. Flesh of non-coated banana presented a brown colour, while silk fibroin-coated fruits preserved a tallow flesh, indication of a decreased ripening rate for the silk fibroin-coated samples.

Climacteric fruits, such as apples, bananas, tomatoes, ripen through ethylene production and increased cell respiration. The climacteric event is associated with changes in fruit colour and with the production of sugar in the extracellular space. To evaluate the efficacy of a silk-based climacteric fruit coating, the ripening of bananas was evaluated by comparing silk coated and non-coated fruits (Fig. 4). Fruits were stored at 22 °C and 38% relative humidity (RH) as received (*No coating*) and after coating with water-annealed treated silk fibroin materials (t_WA_ =D 12 h, *Silk fibroin coating*). Bananas were hung from their respective stem throughout the whole experiment. Time-lapse photography of banana ripening indicated that silk coating decreased the ripening rate. The beta-sheet content of the silk fibroin used to coat the climacteric fruit did not affect fruit ripening (data not shown). Investigation of silk-coated banana firmness showed that the coating decreased fruit ripening, when compared to uncoated control at day 9 after coating. In addition, morphological analysis of the flesh of non-coated and coated bananas at day 9 post silk coating-treatment revealed a more preserved fruit when the silk coating was applied. Flesh of non-coated bananas presented a brown colour, while silk-coated fruits preserved a tallow flesh, indication of a decreased ripening rate within the silk-coated sample.

In summary, we have demonstrated that silk fibroin is an effective, water-based coating to enhance freshness of perishable food. The water-based processing and comestible nature of this material makes the approach a promising alternative for preservation with a naturally derived material. Silk polymorphism may be used to tailor the properties of the coating, affecting the interplay between silk fibroin and water evaporation and food decay. The ability to consume silk or enhance coatings with stabile biomacromolecules further adds utility for functional coatings that could be envisioned to add therapeutic function to consumable goods without resorting to complex chemistries, while preforming the preservation function.

## Methods

### Silk Fibroin Regeneration

Cocoon from *Bombyx Mori* were used as source of fibroin. Extraction of silk fibroin was achieved by standard degumming process, which involved boiling (t =D 30 minutes) 2.5 g of chopped silk cocoons per litre of 0.02 M sodium carbonate solution. SF was then solubilized in 9.3 M lithium bromide for 4 hours in a 60 °C oven. The chaotropic salt was subsequently removed through dialysis (3.5 kDa MWCO) against Milli-Q water for a total of 72 hours, yielding an 8% (w/v) silk fibroin suspension. The resulting SF suspension was then purified by centrifugation at 9000 rpm (~12,700 *g*) over two 25 minute-long periods, at a constant of 4 °C. The final concentration of SF suspension was then adjusted to 5 wt% by adding MilliQ water.

### Strawberry dip coating

For experiments run during the summer, freshly picked New England native strawberries (Wilson Farms, Lexington, MA) were dip coated for 1, 2 and 4 times (namely D1, D2 and D4) in a 60 mm deep silk suspension at 4 °C ensuring that the whole surface of the strawberries and of their calyx and epicalyx were exposed to the suspension. The dipping step(s) last for 10 s each and then strawberries were dried by hanging them from the peduncle for 4 hours at 22 °C, 38% RH. For experiments conducted off-season, strawberries were bought from a local store (Whole Foods, Medford, MA).

### Banana dip coating

Bananas (Del Monte) green in colour were bought from a local store (Whole Foods, Medford, MA) and then dip-coated with silk fibroin as aforementioned.

### Coating crystallization

Silk fibroin coating crystallization (i.e. enhancement of beta-sheet content) was obtained through exposure of coated strawberries to water vapours under vacuum (namely water annealing) at 22 °C, according to previously developed protocols^26^. The choice of operating at room temperature has been dictated by the possible detrimental effects on the fruit quality upon exposure to high temperatures (T >D 40 °C) for prolonged time (t >D 30 minutes). Exposure time was set to 0 s, 1 hours, 6 hours and 12 hours. To longer water annealing post-processing corresponded an increase in the protein beta-sheet content (Table 1), as previously reported^26^.

Attenuated Total Reflectance Fourier Transform Infrared Spectroscopy (ATR-FTIR) was used to evaluate the relative content of beta-sheet structures in silk fibroin materials. FTIR spectroscopy scans were taken on a Jasco FTIR 6200(Easton, MD) spectrometer with attached ATR detector. A total of 64 scans at a resolution of 4.0 cm^−1^ were co-added to produce spectra ranging from 400–4000 cm^−1^. A cosine apodization was simultaneously applied by the software. From these scans, the Amide III region (1200–1350 cm^−1^) was selected for its sensitivity to protein secondary structure and lack of sensitive to water content. Amide III curves were normalized and baseline corrected, and then fit to 12 Gaussian curves. Bands corresponding to beta-sheet secondary structure motifs were then added to give a relative value for the beta-sheet content of the films.

### Evaluation of strawberry freshness

The effect of different silk fibroin coating steps and of water annealing times (i.e. beta-sheet content) on strawberry freshness was evaluated morphologically and gravimetrically. Changes in strawberries colour and shape were evaluated through time-lapse photography. Gravimetric analysis of berries as received in the laboratory, after dip coating and at days 1, 3, 5, 7 and 14 was evaluated with a standard laboratory balance (Mettler Toledo MS204S). Berries weight was calculated as an average of three measurements.

### Water vapour permeability of silk fibroin films

WVP values were determined using the method reported in ASTM E96-E96M^38^, with some modifications^39^,^40^. Circular silk fibroin films (Ø =D 16 mm, n =D 5) of increasing beta-sheet content (36 ±D 4%, 48 ±D 3, 58 ±D 4) were obtained by modulating the exposure to water-annealing vapours at 22 °C, following a previously published experimental procedure, to investigate silk fibroin materials with beta-sheet content comparable to the fibroin coatings^26^. Silk fibroin films were sealed with lubricant grease over the circular opening (Ø =D 14 mm) of custom-made plastic test cups that were stored in a desiccator at 22 °C. A sodium chloride saturated solution was placed in the desiccator (75% RH) while anhydrous calcium chloride pellets were positioned on the bottom of the test cups (0% RH). The difference in RH corresponded to a water vapour partial pressure of 1753.55 Pa. Water vapour permeability was then determined as:


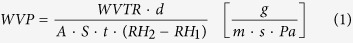


where the water vapour transmission rate (WVTR) was calculated by the linear regression (r^2^ >D 0.98) between the weight gain of the test cups and time, d is the thickness of the silk fibroin samples, A is the area of the circular opening in the test cups, S is the the saturation vapour pressure of water, 2645 Pa at 22 °C, RH_1_ is RH in the desiccator and RH_2_ is RH in the test cup.

### Measurement of oxygen diffusion in silk fibroin films

The effective oxygen diffusion coefficients were measured in silk fibroin films (n =D 5) of increasing crystallinity (23 ±D 5%, 36 ±D 4%, 48 ±D 3, 58 ±D 4) using a conventional diffusion system - Microx TX3 Microsensor Oxygen Meter (Presens, Germany) equipped with an Oxygen Microptode (Presens, Germany) and a PermeGear sealed, water-jacked, gas chamber, as previously described^41^,^42^. The system consisted of two compartments that contained known (measured) concentrations of oxygen, separated by the silk film of interest. The opening between the two chambers had an area of 2.75 cm^2^. Silk fibroin film thickness was measured with a micrometer (n =D 7). Before use, a two-point calibration was performed according to manufacturer protocols using an oxygen-free environment (sodium sulphite) and an air-saturated environment (water vapour). The average of the initial and final oxygen concentration readings was used for diffusivity data analysis. Oxygen concentrations were measured at 10 minutes intervals and each silk fibroin film was tested 3 times consecutively.

The effective oxygen diffusion coefficient was calculated as previously reported^41^,^42^. In brief, under the assumptions of (i) well-mixed fluid in the diffusion system, (ii) no oxygen consumption, (iii) linear oxygen concentration across the tested silk films and (iv) instantaneous steady state, the mass of oxygen in the receiving chamber is described by:


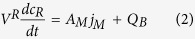



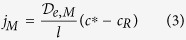



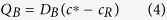


where Equation 2 describes the change of the oxygen concentration (c_R_) as a function of time (t) in the receiving chamber of volume V_R_ through a membrane of area A_M_ and with a flux j_M_. Q_B_ represents the experimental baseline input rate of oxygen. Equation 3 describes the flux of oxygen j_M_ through the semipermeable membrane, where D_e,M_ is the effective membrane oxygen diffusion coefficient, *l* is the membrane thickness and C_D_ is the concentration of dissolved oxygen in the donor chamber, which is constant. Equation 4 describes the experimental baseline flux, which is assumed to be linearly proportional to D_B_, the experimental baseline volumetric diffusion factor. The parameter c*, is the maximum amount of oxygen in solution, which is equal to c_D_ measured in the donor chamber. Equation 4 can then be re-written as:


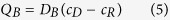


The combination of Equations 2, 4 and 5 gives:


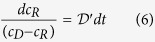


in which


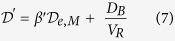


and


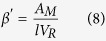


where 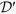 is the diffusion factor and 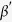 is the effective membrane characteristic geometric constant.

The following initial conditions apply: at t =D 0, c_R_ =D c_R0_. Thus, integrating Equation 6 yields:


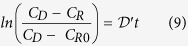


Indeed, the diffusion factor 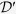
was calculated for each dataset from the slope of the experimental logarithmic concentration data versus time. The baseline volumetric diffusion factor (D_B_) is determined by replacing silk film samples with an oxygen impermeable barrier (i.e. rubber-made stopper). Because it is assumed that in this case D_e,M_ =D 0, then Equation 9 becomes:


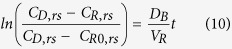


where the suffix *rs* indicates the use of a rubber-made stopper instead of a silk fibroin membrane. The effective silk film oxygen diffusion coefficient D_e,M_ is then calculated as:


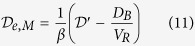


where D_B_ is obtained by applying Equation 10 when the baseline effect is investigated. As baseline effect is independent to the membrane used, the measured D_B_ and 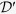 may be used to calculate 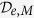 from Equation 11.

### Measurement of strawberry respiration rate

A previously published method was followed to measure the respiration rate of strawberries. In brief, strawberry samples (around 100 g, n =D 3) were placed in a 1 L hermetic glass jars with a septum in the lid for sampling gas at different sampling times, over period of 36 hours. The jars were stored at ambient temperature of 22 °C and RH =D 38%. Gas sampling was carried out every 30 minutes for the first 5 hours, every 90 minutes till the 12th hour and every 180 minutes for the remaining 24 hours with a needle probe. Three replicates were performed for each coating treatment. The respiration rate was calculated by using the following equation:


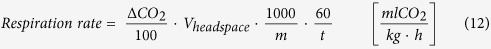


where m is mass of strawberry, V_headspace_ is the empty volume of the hermetic jar [ml], ΔDCO_2_ is the difference between the initial and final concentration of CO_2_ and t is sampling time [min].

### Measurement of strawberry firmness

The firmness of silk fibroin uncoated and coated strawberries was measured at 22 °C and RH =D 38% through a puncture test using an Instron uniaxial system equipped with a 10N load cell. Strawberry firmness was evaluated as a function of storage time (as received, days 1, 3 and 7) and silk fibroin crystallinity degree. The test was performed according to a previously reported protocol^43^. In brief, a 5 mm diameter stainless steel rod with a flat end was used as probe to penetrate the strawberries analysed. The maximum penetration force (N) was defined as the maximum force required pushing the probe into the strawberries (n =D 5) to a depth of 8 mm at a cross-head speed of 1 mm/s.

### Statistics

All data were collected for n =D 5, unless differently specified in the text. Statistical significance of the measurements was evaluated via one-way ANOVA test with Tukey mean analysis.

## Additional Information

**How to cite this article**: Marelli, B. *et al.* Silk Fibroin as Edible Coating for Perishable Food Preservation. *Sci. Rep.* **6**, 25263; doi: 10.1038/srep25263 (2016).

## Supplementary Material

Supplementary Information

## Figures and Tables

**Table 1:** Characterization of edible silk fibroin coating thickness as a function of dip coating steps and of relative beta-sheet content as a function of exposure time to water annealing.

Number of dip coating processes (x) in silk fibroin suspension [Dx]	Thickness [μDm]
D1	27 ±D 8
D2	32 ±D 7
D4	35 ±D 8
Exposure time to water annealing post-processing [hours]	Silk fibroin coating crystallinity [relative content of beta-sheet structures, %]
0	23 ±D 2
1	36 ±D 3
6	48 ±D 4
12	58 ±D 5

No statistically significant difference was found in the coating thickness for increasing number of dip coating steps. The relative beta sheet content of silk fibroin coating was calculated with a previously reported methodology based on the quantification of the relative beta sheets structures in the Amide III absorbance peak collected with ATR-FTIR spectroscopic analysis.

**Table 2:** Calculated water vapour permeability in silk fibroin films of increasing beta-sheet content (ΔRH = 75%).

Silk fibroin edible coating beta-sheets content	Water vapour permeability
(g m^−1^s^−1^ Pa^−1^)	
36%	7.93 · 10^−9^
48%	5.37 · 10^−10^
58%	6.49 · 10^−11^
