# Supplementary material for: Silk Fibroin as Edible Coating for Perishable Food Preservation
Source: Sci Rep. 2016 May 6;6:25263. doi: 10.1038/srep25263 (PMC4858704; doi:10.1038/srep25263)
Supplement: Supplementary Information [file srep25263-s1.pdf]

Supporting Information

Silk Fibroin as Edible Coating for Perishable Food Preservation

*Benedetto Marelli, Mark Brenckle, David L. Kaplan and Fiorenzo G. Omenetto**

B. Marelli, M. Brenckle, D.L. Kaplan and F. G. Omenetto*
Department of Biomedical Engineering
Tufts University
4 Colby St., Medford, MA, 02155, USA
E-mail: Fiorenzo.Omenetto@tufts.edu

F. G. Omenetto
Department of Physics
Tufts University
4 Colby St., Medford, MA 02155

Keywords: fruit, preservation, coating, silk, food

Table S1 ICP-MS trace element analysis of water-based silk fibroin suspensions. Values are in part per million. ND=non detected. *n*=5. As comparison, toxicity threshold values for drinking water[1],[2] were reported in the fourth column of the table.

Element	Silk fibroin (2 wt%)	Detection Limit	Toxicity threshold in drinking water[1]
Calcium	2.2±0.8	0.3	150
Chromium	ND	0.01	0.05
Copper	0.013±0.03	0.002	2
Gold	ND	0.002	Not regulated. Generally considered non toxic
Iron	0.07±0.02	0.05	2 (suggested value[2])
Lead	ND	0.002	0.01
Lithium	0.387±0.09	0.006	5 (Not regulated by WHO. Suggested value[2])
Magnesium	0.16±0.01	0.06	150
Manganese	ND	0.002	0.4
Nickel	0.006±0.001	0.002	0.07
Potassium	1.8±0.1	0.5	12 (Not regulated by WHO. Suggested value[2])
Silver	ND	0.002	0.002
Sodium	113.5±0.5	0.5	200 (Not regulated by WHO. Suggested value for taste)
Tin	ND	0.002	1000 (Not regulated by WHO. Suggested value[2] as not absorbed by the body)
Zinc	0.05±2	0.03	5

Table S2 Calculated diffusion coefficients for water mass transport experiments. *k* and *n* are fitting parameters while *D* is the diffusion coefficient.

Silk fibroin beta-sheet content	k	n	D (cm^2/s)
36%	0.25 ± 0.048	0.42 ± 0.04	5.79×10^{-6}
48%	0.19 ± 0.039	0.48 ± 0.03	3.21×10^{-6}
58%	0.16± 0.027	0.55 ± 0.02	1.05×10^{-6}

Table S3 Calculated effective silk fibroin O_2 diffusion coefficient for gas transport experiments.

Silk fibroin beta-sheet content	Film thickness μm	Effective membrane O_2 diffusion coefficient [$D_{e,M}$] (steady state) 10^{-11}• (cm^2/s)
23%	81±4	83.9±7.2
36%	78±3	67.9±3.5
48%	76±4	5.5±1.4
58%	73±2	1.9±0.3

Figure S1

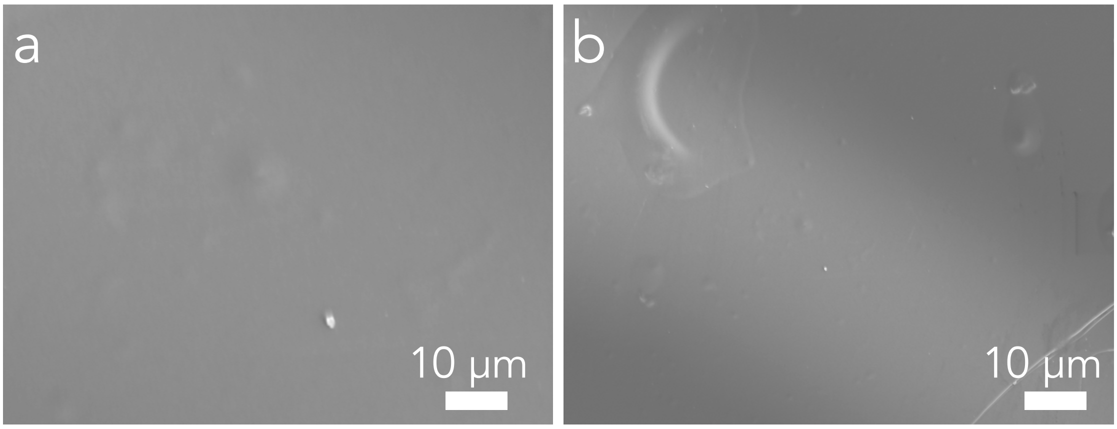

Figure S1. Scanning electron microscopy of silk fibroin membranes a) with 23% beta-sheet structures (i.e. before water annealing process and b) with 58% beta-sheets structures (i.e. at 12 hours of water annealing). The micrographs depict no changes in the morphology of the membranes at the microscale.

Figure S2

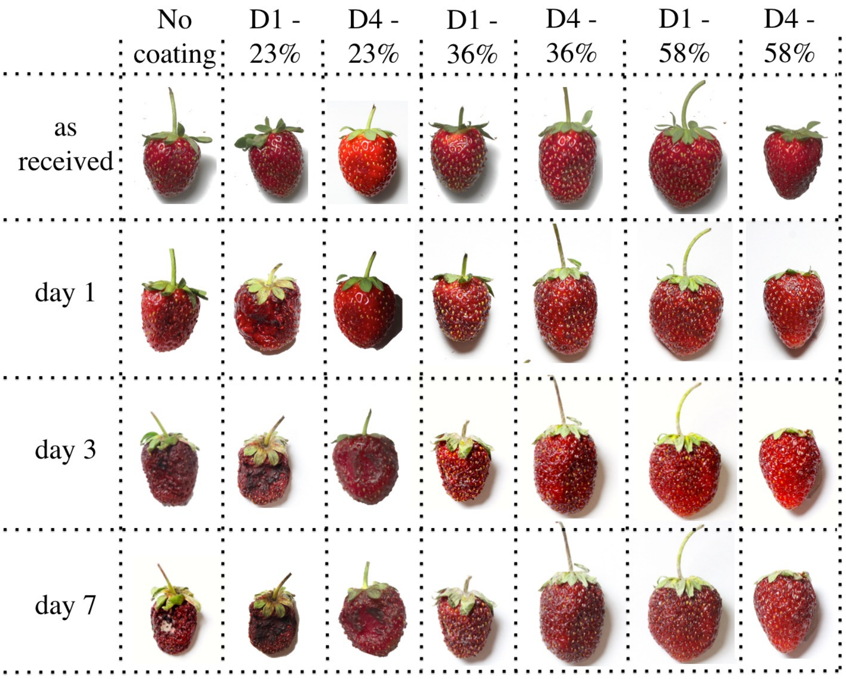

Figure S2. Time-lapse of strawberries ripening. Berries were stored at 22˚C and 38% RH as received (*no coating*) and after coating with silk fibroin suspension (*Dx-xx%*). *Dx* stands for 'x' dip coating steps. *xx%* stands for relative content of beta-sheets structures in the silk coating. The

amount of beta-sheets was modulated by varying the exposure time to water annealing process. Thus, D1-58% refers to strawberries that were dip coated 1 time in silk fibroin solution and that were exposed to a water annealing process that yielded 58% of beta-sheets in the protein structure.

Reference

[1] FAO. Global food losses and food waste. (2011) Available at http://www.fao.org/docrep/014/mb060e/mb060e.pdf (Accessed: 8th March 2016).

[2] J. DeZuane, in *Handbook of Drinking Water Quality*, Wiley, 49-147 (John Wiley & Sons, Inc., 1997).
